# A Simple, Low-Cost Implant for Reliable Diaphragm EMG Recordings in Awake, Behaving Rats

**DOI:** 10.1523/ENEURO.0444-24.2025

**Published:** 2025-02-18

**Authors:** Taylor C. Holmes, Jesus D. Penaloza-Aponte, Alyssa R. Mickle, Rachel L. Nosacka, Erica A. Dale, Kristi A. Streeter

**Affiliations:** ^1^Exercise and Rehabilitation Science Program, Department of Physical Therapy, Marquette University, Milwaukee, Wisconsin 53233; ^2^Department of Neuroscience, University of Florida, Gainesville, Florida 32610; ^3^Breathing Research and Therapeutics Center, University of Florida, Gainesville, Florida 32610; ^4^McKnight Brain Institute, University of Florida, Gainesville, Florida; ^5^Departments of Pediatrics, University of Florida, Gainesville, Florida 32610; ^6^Physiology and Aging, University of Florida, Gainesville, Florida 32610

**Keywords:** breathing, chronic recordings, diaphragm, electromyography, respiratory muscle

## Abstract

Breathing is a complex neuromuscular process vital to sustain life. In preclinical animal models, the study of respiratory motor control is primarily accomplished through neurophysiologic recordings and functional measurements of respiratory output. Neurophysiologic recordings that target neural or muscular output via direct nerve recordings or respiratory muscle electromyography (EMG) are commonly collected during anesthetized conditions. While offering tight control of experimental preparations, the use of anesthesia results in respiratory depression, may impact cardiovascular control, eliminates the potential to record volitional nonventilatory behaviors, and can limit translation. Since the diaphragm is a unique muscle which is rhythmically active and difficult to access, placing diaphragm EMGs to collect chronic recordings in awake animals is technically challenging. Here, we describe methods for fabricating and implanting indwelling diaphragm EMG electrodes to enable recordings from awake rodents for longitudinal studies. These electrodes are relatively easy and quick to produce (∼1 h), are affordable, and provide high-quality and reproducible diaphragm signals using a tethered system that allows animals to *ad libitum* behave. This system is also designed to work in conjunction with whole-body plethysmography to facilitate simultaneous recordings of diaphragm EMG and ventilation. We include detailed instructions and considerations for electrode fabrication and surgical implantation. We also provide a brief discussion on data acquisition, material considerations for implant fabrication, and the physiological implications of the diaphragm EMG signal.

## Significance Statement

Investigations of respiratory neuromuscular output have frequently involved the diaphragm muscle, given its role as the main inspiratory muscle in mammals. While anesthetized preparations are fundamental to our understanding of respiratory neuromuscular control, using awake, *ad libitum* behaving models enhances translatability, particularly when developing and evaluating therapeutic strategies in conditions that result in respiratory pathology and impaired breathing. Here, we describe an affordable and easy-to-produce electromyography implant that enables stable recordings of the diaphragm output in awake, *ad libitum* behaving rodents over long-term experimental protocols and offer a brief commentary on data acquisition and analysis of these signals.

## Introduction

Breathing is a complex, rhythmic motor process involving the coordination of many muscles. The diaphragm is considered the main inspiratory muscle in mammals and also plays a role in other critical nonventilatory behaviors such as cough, sigh, and sneeze (Sieck and Fournier, 1989; Mantilla et al., 2011; Fogarty et al., 2018). The dome-shaped structure of the diaphragm can be divided into costal (ventral), crural (dorsal), and sternal regions (Kocjan et al., 2017). Although all regions of the diaphragm participate in respiratory activity; the costal region is thought to contribute most to normal, quiet breathing (Pickering and Jones, 2002; Nason et al., 2012; Merrell and Kardon, 2013). The diaphragm is controlled by phrenic motor neurons found in the cervical spinal cord, which transmit signals via the phrenic nerve (Ricoy et al., 2019; Fuller et al., 2022). The phrenic nerves insert into the costal diaphragm and provide rhythmic neural drive to facilitate diaphragm contraction (Nason et al., 2012; Fuller et al., 2022).

Preclinical work has investigated diaphragm activity through implanted electromyography (EMG) electrodes with the majority of studies recording diaphragm EMG output under anesthetized conditions (Dow et al., 2006, 2009; Mantilla et al., 2010, 2011, 2013; Seven et al., 2014; Lee and Hsu, 2017; Rana et al., 2017, 2020; Jimenez-Ruiz et al., 2018; Popp et al., 2023; Mickle et al., 2024; Khurram et al., 2024a,b). More recent studies from our laboratories and others have examined diaphragm activity in awake, *ad libitum* behaving rats (Navarrete-Opazo and Mitchell, 2014; Alvarez-Argote et al., 2016; Bezdudnaya et al., 2018; Jimenez-Ruiz et al., 2018; Rana et al., 2021; Malone et al., 2022; Khurram et al., 2023, 2024a,b; Holmes et al., 2024). While anesthesia offers tight control of experimental preparations, its use can limit the translational nature of the experimental findings. Specifically, anesthesia can suppress central respiratory drive (Mills, 2018; Ballók et al., 2023) and can alter important variables such as cardiovascular and respiratory pattern and reflexes (Hunter and Milsom, 1998). Recording diaphragm EMG output in awake conditions allows for longitudinal studies of the diaphragm muscle during ventilatory behaviors without the confounding effects of anesthesia on neuromotor control. It also allows for nonventilatory behaviors to be recorded including sniffing and sighing. However, given the location of the diaphragm within the body cavity, its rhythmic activity, and the associated risk of pneumothorax, designing and implanting EMGs into the diaphragm muscle present distinct challenges. To address these issues, we designed a low-cost, easy-to-produce EMG method that effectively overcomes these obstacles and achieves successful outcomes (Malone et al., 2022; Holmes et al., 2024). Here, we detail the methods that enable chronic recordings of bilateral diaphragm EMG activity in awake, *ad libitum* behaving rodents. We follow with a brief discussion on data acquisition, interpretation, and equipment considerations for device fabrication.

## Materials and Methods

### Animals

All animal procedures were performed in accordance with the Institutional Animal Care and Use Committee at Marquette University (Protocol No. AR-4198) and University of Florida and conformed to the Society for Neuroscience Policies on the Use of Animals in Neuroscience Research. Here we provide instructions to make and implant diaphragm EMGs from our experience with *n* = 69 male and *n* = 42 female Sprague Dawley rats. We provide new data [coefficient of variation (CV) for peak EMG, signal-to-noise ratio, and representative images of EMG output] from animals (*n* = 6 male and *n* = 7 female) in which EMG signals were recorded for 8 weeks after implant included in our previously published study (Holmes et al., 2024). Additionally, we include new data (signal-to-noise and representative images of EMG) from animals (*n* = 6 males) in which EMG signals were recorded for 40 weeks after implant that are included in a manuscript currently in press. We also report surgical and postoperative complications from *n* = 105 of these animals.

### Data analysis and statistics

Signal-to-noise ratio was calculated from the integrated channel of diaphragm EMG measurements over a five-breath window of quiet breathing. The signal-to-noise ratio represents the peak amplitude during the inspiratory burst compared with the peak amplitude between each inspiratory burst. CV was determined by taking the average peak amplitude over a 60 s window of quiet breathing across early (4–5 d after implantation) and chronic (8 weeks postimplantation) timepoints. Paired *t* tests were used to compare the signal-to-noise ratio across 8 and 40 week timelines. The significance level was set to *p* < 0.05, and all data are presented as standard error of the mean (SEM).

### Fabrication of the electrodes

**Figure 1.**
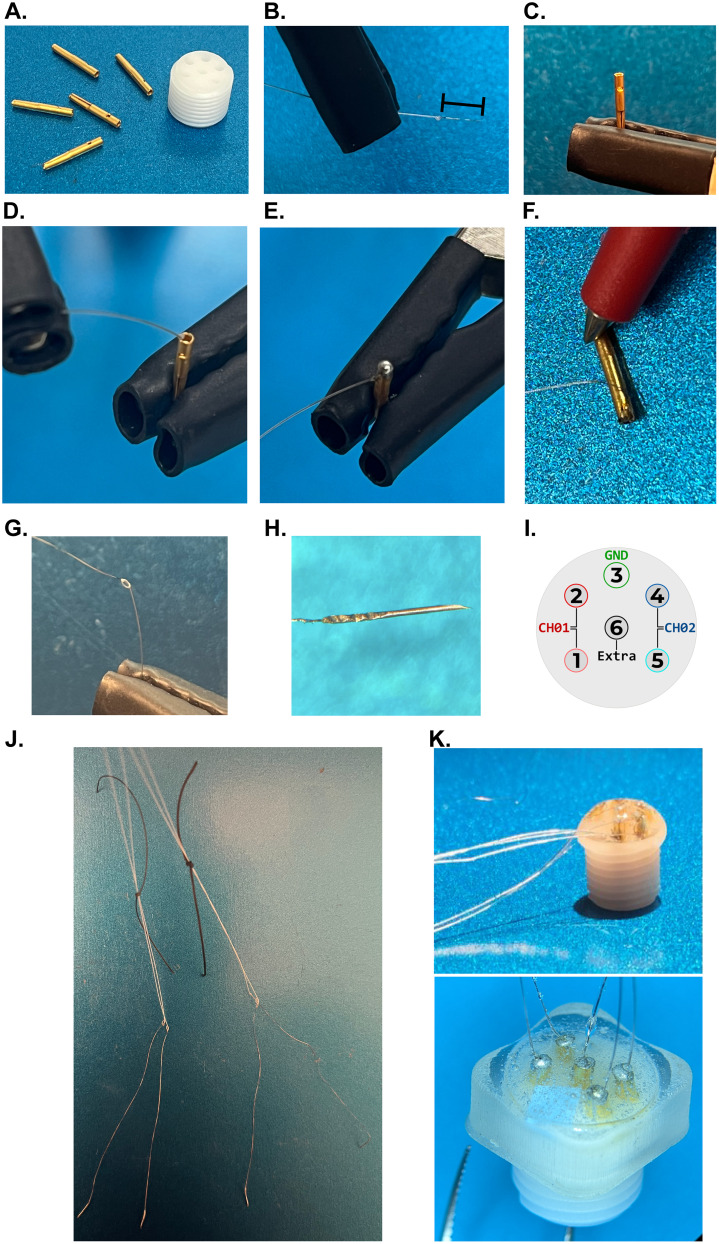
Diaphragm EMG electrode fabrication and assembly. ***A***, Each EMG implant is composed of one plastic pedestal and five or six (if using an extra/backup electrode) gold pins. ***B***, Deinsulate ∼2 mm of wire which will interface with a gold pin. Deinsulated portion of the wire is denoted with the bracket. ***C***, Use the helping hands to hold the gold pin in an upright position. ***D***, With a second helping hand, place the deinsulated end of the wire inside the gold pin. ***E***, Solder the wire and gold pin with enough solder to fill the pin, but not spill out of the pocket. ***F***, Use a multimeter to measure the resistance between gold pins on the same wire. Repeat for all recording and ground wires. Cut each wire in half. ***G***, Tie an anchor knot on each recording wire opposite of the gold pin. Deinsulate the wire from the anchor knot to the distal end. ***H***, Crimp a 31 G insulin needle onto the wire using a flat-blade screwdriver. ***I***, Place each pin into the pedestal according to the configuration. We utilize Pinholes 1 and 2 (CH01) for the left hemidiaphragm, Pinholes 4 and 5 (CH02) for the right hemidiaphragm, Pinhole 3 for the ground, and Pinhole 6 for the extra, backup electrode (if used). It is vital to maintain a consistent configuration among produced implants to ensure correct identification of paired electrodes. ***J***, Once assembled, tie suture around the pairs of electrodes to identify during surgery. Wires from electrodes in Pinholes 1 and 2 (CH01/left) will be identified with a size 0 suture; wires from electrodes in Pinholes 4 and 5 (CH02/right) will be identified with a size 3.0 suture. All recording electrodes will then be tied together with a larger size 0 suture to assist when tunneling the wire under the skin. ***K***, Completed implant is shown with the cured epoxy both with and without using the 3-D–printed headcap holder. If using partial-thickness electrodes, steps found in subpanels ***G*** and ***H*** will not be performed.

Materials needed for the fabrication of the diaphragm EMG implant are shown in Table 1. A schematic of electrode fabrication and assembly can be found in Figure 1 and is referenced throughout the protocol. In total, it requires ∼1 h to build one implant and to apply the epoxy.

1. Prepare and clean the individual pieces of the electrode (Fig. 1*A*).
  - Pass the top surface of the pedestal over the sandpaper 2–3 times to aide mechanical bonding at later steps.
  - Spray the gold pins and plastic pedestal with 70% alcohol to remove any residual oils and promote proper solder and epoxy binding. Let the materials dry completely before proceeding.
2. Cut three 32 cm recording wires and one 16 cm grounding wire.
  - Four recording electrodes will be implanted into the diaphragm muscle. A single ground wire will be used for this implant.
  - Each plastic pedestal has six open pinholes. Implants can be constructed using five gold pins (four electrodes, one ground) or six gold pins (four electrodes, one backup electrode, one ground). If utilizing five gold pins, cover the additional, open pinhole with a small piece of tape.
3. Deinsulate ∼2 mm of the perfluoroalkoxy (PFA) coating from each end of all wires using either a scalpel blade or an open flame (Fig. 1*B*).
  - Deinsulating wires with an open flame can leave residue on the metal wire and may affect connectivity and soldering connections at later steps.
4. Prepare the gold pin and wire connections.
  - Brush the deinsulated portion of the wire and the edge of the gold pin with a flux pen.
  - Avoid touching the end of the wire or the pin with ungloved hands.
5. Solder the wire to the gold pin.
  - Using a heat-resistant silicone cover on the alligator clip from the helping hands to reduce damage to the pin and increase friction between the pin and the helping hands to improve stability, hold the gold pin in an upright position (Fig. 1*C*).
  - Use an additional silicone-covered alligator clip to hold the wire.
  - Place the deinsulated portion of the wire inside the gold pin (Fig. 1*D*).
  - Use enough solder to fill the pin, but not so much that it spills out.
  - Once completed, cover the top of the pin with solder to create a “dome” to reinforce the wire–pin structure by briefly touching the top of the pin with the solder iron (Fig. 1*E*).
  - Repeat until all wires have gold pins at each end.
  - Use a multimeter to measure the resistance between the gold-plated pins on each recording wire (Fig. 1*F*).
    - Expected resistance of recording wire is <80 Ω. Expected resistance of ground wire id <40 Ω.
      1. Note: resistance has a direct relation to the length of the wire (i.e., Higher resistance for longer wires; *R* = *ρ* * (*l*/*A*) where *R* is resistance, *ρ* is the resistivity of the wire material, *l* is the length of the wire in meters, and *A* is the cross-sectional area of the wire (in square meters).
  - Cut each wire in half (∼16 cm for recording wire, ∼8 cm for ground wire).
    - The length of the wire can be extended to match the size of the animal. We have had success with electrodes up to a length of 25 cm (enough for the wire to reach from the headcap to the diaphragm with additional length to allow for movement and growth of the animal). Adjust the initial length of wire in Step 2 as appropriate if wanting to use longer electrodes.
6. Prepare the distal portion of the electrode to interface with the muscle. Electrode placement can be either “full-thickness” or “partial-thickness.” Full-thickness placement penetrates through the diaphragm muscle, while partial-thickness placement is embedded within the diaphragm muscle (details below). We have had success with both full- and partial-thickness placement of electrodes. When electrodes are used for partial-thickness implantation, additional assembly steps at the time of surgical implantation are required.
  - Partial-thickness placement: No additional steps needed at this time.
  - Full-thickness placement: Tie an anchor knot ∼2 in from the distal portion of the recording wire. Deinsulate the wire from the end to the anchor knot (Fig. 1*G*). Remove a 31 G needle from a BD insulin syringe. Thread the deinsulated portion of the wire inside the 31 G needle. Crimp the end of the needle where it meets the wire using a flat-blade screwdriver (Fig. 1*H*).
    - Be sure that the wire is not exiting the needle at the beveled end.
7. Assemble the headcap according to the configuration provided in Figure 1*I*.
  - Identify the pairs of recording electrodes by tying the suture of varied sizes around each pair (Fig. 1*J*).
    - Electrodes which will be placed in the left hemidiaphragm can be denoted by using a small length of size 0 suture.
    - Electrodes which will be placed in the right hemidiaphragm can be denoted by using a small length of size 3.0 suture.
    - All recording electrodes are grouped by using a longer length of size 0 suture (note: the longer size 0 suture grouping all recording electrodes is not shown in Fig. 1*J* for clarity).
8. Cover the pedestal and gold pins of the implant with clear epoxy for protection using a 1 ml syringe (Fig. 1*K*). Two options are available to facilitate this step:
  - If an SLA printer is available, 3-D print the provided headcap holder (Data Repository 1 https://figshare.com/s/c1ae2d737196e34f6b34).
    - Allow the epoxy to cure for 2–3 min in the syringe before applying over the pedestal. The headcap holder aids in shaping the epoxy which reduces the spillover of the epoxy around the edges of the headcap (Fig. 1*K*, bottom image).
  - If no printer is available, secure the pedestal on the mounting holder so that the soldered gold pins are facing up.
    - Allow the epoxy to cure for ∼10 min in the syringe prior to application. Use a thin wooden dowel to shape the epoxy into a dome structure throughout the remaining cure time (20–30 min; Fig. 1*K*, top image).
  - Allow the epoxy to dry for 24 h.
9. Autoclave the completed implant using settings for dry surgical instruments.

**Table 1.** Materials and equipment required to build the electrodes

Component	Description	Quantity	Catalog #	Company
Sandpaper	Sanding block	1	DSFM-F-ESF-10	3M
Kimtech KimWipes	Delicate task wipers	1	34155	Kimberly-Clark Professional
Gold pins	Six-channel electrode female socket	5 (6 if utilizing an extra, backup electrode)	E363/0	Protech International
Plastic pedestal	Six-channel electrode pedestal	1	MS363	Protech International
Recording wire	Multistranded PFA-coated stainless steel wire	(3) 36 cm sections of wire	793200	A-M Systems
Grounding wire	Stranded stainless steel wire	(1) 16 cm section of wire	AS631	Protech International
Scalpel blade	Size 10 scalpel blade	1		Amazon
Lighter	Pocket-style, classic lighter	1		Amazon
31 G needle	Insulin syringe with ultrafine needle	5	NDC/HRI#: 08290-3249-10	Amazon
Screwdriver	Fine Science Tools Screwdriver Set, Nickel, six total	1 (size 1.8 or 2.3)	NC9468697	Thermo Fisher Scientific
Suture	Braided silk suture (size 0 and 3-0)	1 ea	Size 0: SUT-S 116 Size 3-0: SUT-S 110	Braintree Scientific
Epoxy	Devcon 2-ton epoxy	1	Devcon 31345	Amazon
Multimeter	Digital multimeter	1	AstroAI WH5000A	Amazon
Helping hands	Helping hands platform and clips	1	OF-M4	OMNIFIXO
Solder iron	Digital soldering station	1	FX888D	Amazon
Solder	44 Flux Cored Wire (SN60PB40 3.3%/44)	1	2460400027	Kester
Flux pen	951 Flux-Pen	1	8310000951	Kester
Microscope	Can be either stereo or surgical microscope	1		
Mounting holder	Six-channel mounting holder	1	MH-363	Protech International

### Implantation of diaphragm electrodes

All procedures described below should be performed under aseptic conditions. Equipment needed for surgical implantation can be found in Table 2.

**Figure 2.**
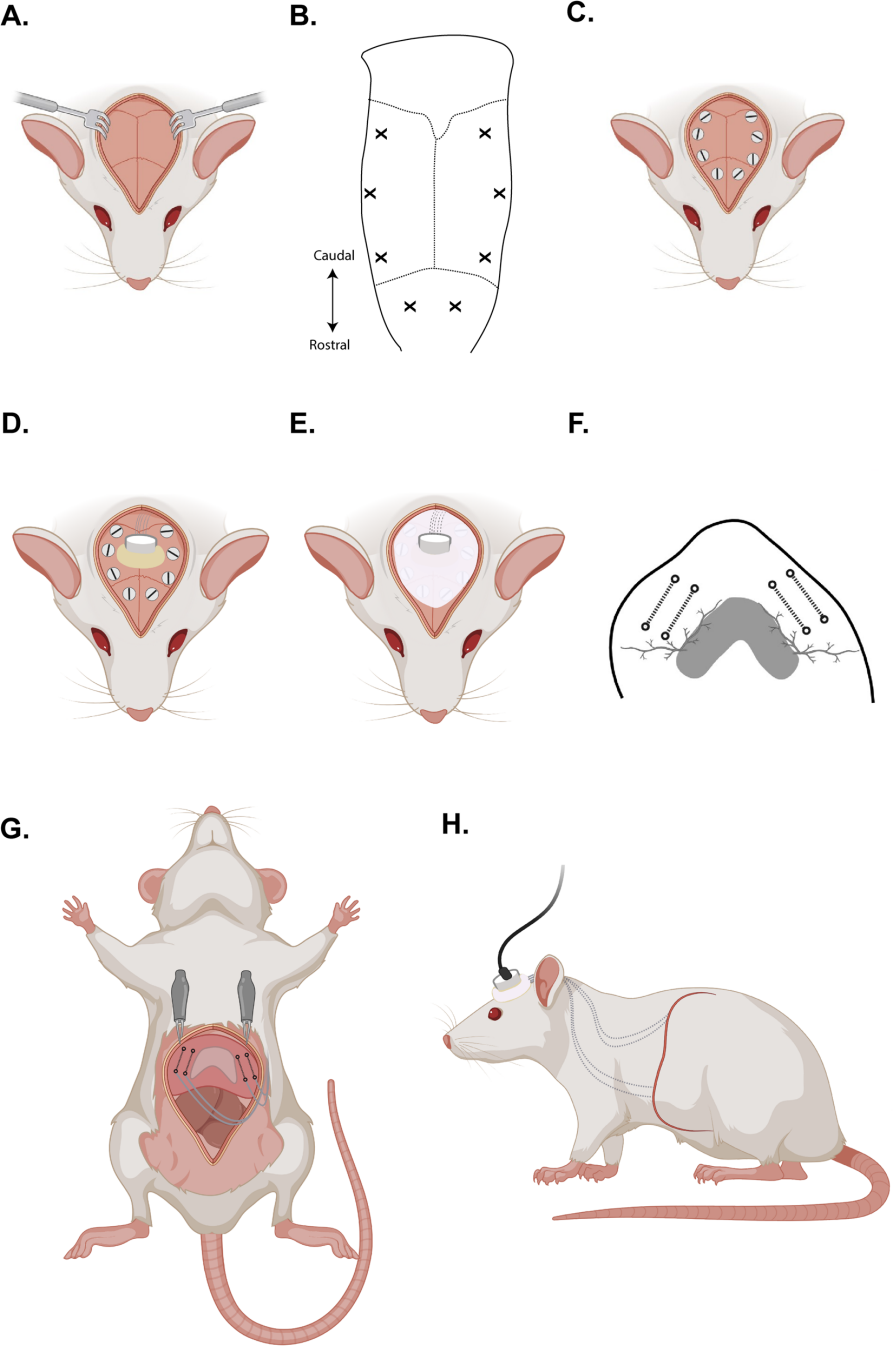
Surgical placement of the diaphragm EMG implant. ***A***, Create a midline incision over the skull, and clear the underlying connective tissue for a clear view of sutures on skull. ***B***, Schematic depiction of location for screw placement. Use a 0.5-mm-diameter burr to drill into the skull at each location marked by “X”. ***C***, Place self-tapping bone screws at each location, leaving the uppermost portion of the screw exposed. ***D***, Once wires are tunneled from skull to abdomen, place implant epoxy-side down onto the skull between the screws. ***E***, Use dental acrylic to secure the implant in place. Dashed lines indicate wires underneath the dental acrylic. ***F***, Using the helping hands, tent the abdomen to view the diaphragm muscle. Insert electrodes at the black X's and advance behind the diaphragm and then back out. Gently pull until the anchor knot is touching the diaphragm and tie a second anchor knot. ***G***, Repeat until two pairs of electrodes are placed in the diaphragm muscle. ***H***, Schematic of the completed diaphragm EMG implant with subcutaneous wires extending from the secured implant to the abdomen and into the diaphragm muscle. Recording cables are plugged into the implant to record the EMG output. Dashed lines show subcutaneous wires from EMG implant to abdomen. Created in BioRender. Streeter (2024) BioRender.com/o298768.

1. Anesthetize animal with isoflurane or other preferred anesthetic per individual institution and protocol guidelines.
  - Once anesthetized, move the animal to the presurgical station and maintain anesthesia in 100% oxygen through a nose cone.
  - Confirm effective sedation via protocol guidelines.
2. Prepare the surgical sites.
  - Shave the dorsal surface of the animal's head and back, beginning caudal from the eyes and extending to the inferior angle of scapulae.
  - Carefully flip the animal and shave the abdomen from the xiphoid process of the sternum to just rostral of the urethral orifice.
  - Move the animal back to prone position being careful to maintain the nose in the nose cone.
  - Clean and disinfect the dorsal surgical area (skull and scapulae) using alternating chlorohexidine and isopropyl alcohol or sterile saline gauze (follow the guidelines of your institution for aseptic practice).
  - Carefully move the animal to the sterile surgical field in the supine position onto a piece of commercially available cling film (Emmer et al., 2019) or alternative sterile surgical wrap. Be sure to avoid contact with the prepped dorsal surgical area.
  - Clean and disinfect the abdominal surgical area using alternating chlorohexidine and isopropyl alcohol gauze or sterile saline gauze. Allow the abdomen to dry and then wrap the cling film or surgical wrap around the animal. This method allows surgeons to flip the animal multiple times while maintaining a sterile field throughout the procedure.
  - For optimal visualization, the animal should be positioned perpendicular to the surgeon (with the tail closest to the surgeon).
3. Return animal to the prone position.
4. Fix the implant to the skull via bone screws and dental acrylic.
  - Make a midline incision over the skull and clear the underlying connective tissue from the skull using scissors and a curette (Fig. 2*A*).
  - To further aide mechanical bonding of the dental acrylic, the skull can be lightly scored in a crisscross pattern by using the tip of the scalpel. This step is optional.
  - Use a 0.5-mm-diameter burr to drill into the skull at specified locations between the bregma and lambda sutures (Fig. 2*B*).
  - Place self-tapping bone screws at each location, leaving the uppermost 1.5 mm of screw exposed above the skull (Fig. 2*C*).
5. Tunnel the wires from the skull to the abdomen using Adson forceps and fine scissors.
  - Start at the base of the skull and create a subcutaneous tunnel angled away from spinal midline toward the left scapula using blunt dissection techniques. Once the tunnel extends beyond the scapula, move the animal into a supine position.
  - Make a midline incision through the skin, but not the abdominal musculature, extending from the xiphoid process to the lower abdomen.
  - Use the Adson forceps and fine scissors to create a subcutaneous tunnel from the rostral portion of the abdominal incision toward the tunnel extending from the skull.
    - For best results, the animal should be placed on their right side to connect the two tunnels.
  - Once complete, advance a Halsted-Mosquito Hemostat through the subcutaneous tunnel from the abdomen to the skull.
  - Use the Halsted-Mosquito Hemostat to grab onto the implant where the suture is tied around all wires (above the knots). Gently pull the recording electrodes from the base of the skull to the abdominal opening. Once the electrodes are through, move the animal back into the prone position.
6. Wrap the ground wire around the bone screws at the skull.
  - The epoxied portion of the implant should be in contact with the exposed skull (Fig. 2*D*) while avoiding putting too much tension on the ground wire.
7. Secure the headcap to the skull by using dental acrylic via a 1 cc syringe and 18 G needle.
  - For best results, dental acrylic should be both underneath and completely around the epoxied pedestal.
  - Allow the dental acrylic to dry (∼5 min) and clean any acrylic from the surrounding tissue.
  - Use suture to close the skin caudal to the implant/headcap if needed.
8. Move the animal back into the supine position for the remaining steps of implantation.
9. Open the abdominal wall along the linea alba to expose the abdominal cavity and view the diaphragm muscle.
  - Use Adson forceps to tent the abdominal musculature away from the abdominal viscera prior to cutting.
  - Open the abdominal cavity from the sternum to the lower abdomen to optimize your viewing window.
  - For partial-thickness placement of electrodes, wires can enter the abdominal cavity via this midline incision or via a lateral approach. For the lateral approach, pierce the abdominal wall away from the midline with a 16 or 18 G needle. Pass the wires through the needle and into the abdominal cavity. Remove the needle by advancing completely through the abdominal wall while being careful not to contact the abdominal viscera. This lateral approach may serve to decrease irritation at the midline abdominal incision once the animal is awake.
    - For full-thickness electrode placement, wires are unable to be passed into the abdominal cavity using the lateral approach since the needle is already crimped onto the distal end of each electrode.
10. Optimize the view of the abdominal cavity by utilizing alligator clips to hold the muscle at each side of the sternum. Be careful not to clip onto the skin as this will make surgical closure difficult at later steps (Fig. 2*E*).
  - Lift the clips away from the body to visualize the abdominal portion of the diaphragm.
  - The liver may obstruct the initial view of the diaphragm muscle and can be carefully moved away from the viewing window with forceps and by cutting the Falciform ligament connecting the liver to the abdominal wall. This step is optional as it may cause more bleeding.
11. Implant pairs of electrodes into the midcostal region of each hemidiaphragm (Fig. 2*F*).
  - Remove the longer length size 0 suture which surrounds all recording electrodes and separate the electrodes for the left hemidiaphragm from those for the right hemidiaphragm. Cover one pair with sterile gauze to prevent the wires from getting mixed up. Remove the suture surrounding the pair that you will implant first.
  - For full-thickness placement of electrodes, hold the crimped needle at its base using the Halstead-Mosquito Hemostat.
    - Orient the beveled side of the needle to be nearly parallel to the diaphragm muscle wall. Holding it close to the base will allow for a larger portion of the needle to pass behind the diaphragm muscle.
    - Insert the needle through the dorsal part of the midcostal diaphragm (ventral to the phrenic artery). Utilize the diaphragm contraction to assist with insertion and advancement of the needle through the muscular wall to avoid excessive force on the muscle. Advance the needle toward the ventral aspect of the animal with the contraction. The needle should span a distance of ∼3–5 mm prior to passing back through the diaphragm muscle to the abdominal cavity (Fig. 2*G*, dashed lines). Note: do not let go of the needle base until the needle tip has passed back through the diaphragm.
    - Gently pull the needle until the anchor knot contacts the diaphragm muscle. Tie a second knot on the free end of the wire to secure the electrode against the muscle. Cut and discard the excess wire and needle.
    - Repeat this process to place a pair of electrodes on each hemidiaphragm in a parallel alignment with ∼4–5 mm between electrodes.
    - If you included a fifth recording electrode and do not need it for surgery, cut the additional recording electrode above the anchor knot and fold the remaining wire into the abdominal cavity.
  - For placement of partial-thickness electrodes, detach an insulin needle and bend the tip of the needle to a 45–60° angle.
    - With the concave aspect of the bent needle facing the surgeon, gently insert the needle into the superficial layer of the diaphragm muscle. Be careful not to completely penetrate the muscular wall.
    - Advance the needle toward the ventral aspect of the animal for ∼2 mm and push the tip of the needle back through the superficial diaphragm muscle layer into the abdominal cavity. The needle will stay in the diaphragm muscle with both ends exposed to the abdominal cavity.
    - Using Castroviejo needle holders, grasp the distal tip of the electrode/wire and insert into the beveled tip of the needle. Advance the wire so that it exits at the base of the needle.
    - Once the wire has extended through the length of the needle, gently hold the needle at its base and remove from the diaphragm. The electrode wire will stay within the muscle in the same path made by the needle.
    - Pull the electrode toward the opening of the abdominal cavity, and use a scalpel blade to expose the distal 0.5 cm of the coated electrode. Then, tie three square knots using nondissolvable 6″ suture at the end of the electrode.
    - Pull the electrode in reverse until the suture knots reach the diaphragm muscle.
    - Either use tissue glue or tie another knot at the point of entry of the wire in order to secure.
    - Repeat this process to have a pair of electrodes on each hemidiaphragm in a parallel alignment with ∼4–5 mm between electrodes.
12. Carefully close the abdominal musculature and skin using sterile suture.
  - We have had success closing the muscle layers of the abdomen using interrupted sutures and closing the skin with subcuticular absorbable suture.
13. Remove anesthesia and provide postoperative pain medications and antibiotics according to institutional guidelines. Schematic depictions of the implant can be found in Figure 2, *G* and *H*.

**Table 2.** Equipment and tools needed to implant the diaphragm EMGs

Component	Description	Quantity	Catalog #	Company
Chlorhexidine scrub	ChlorHex-Q Scrub	1	VINV-CLOR-SCRB	Vedco
Sterile saline	Saline solution 0.9%—sterile	1	07-841-4550	Patterson Veterinary
Scalpel handle	Scalpel Handle #3	1	10003-12	Fine Science Tools
Scalpel blade	Size 10	1		
Scissors	Fine scissors	1	14558-11	Fine Science Tools
Forceps	Adson Forceps	1	11006-12	Fine Science Tools
Curette	Micro Curette	1	10082-15	Fine Science Tools
Retractors	Alm Retractors	1	17008-07	Fine Science Tools
Drill	Dremel Micro	1	8050	Dremel
Collet for drill	3/32″ Rotary Tool Collet (Dremel 4485)	1		Amazon
Drill bit	Burrs for Micro Drill	1	19007-07	Fine Science Tools
Bone screws	Self-tapping bone screws	1	19010-10	Fine Science Tools
Screw forceps	Screw forceps	1	26100-00	Fine Science Tools
Screwdriver	Fine Science Tools Screwdriver Set, Nickel, six total	1 (size 1.5 or 1.8)	NC9468697	Thermo Fisher Scientific
Dental acrylic	Dental acrylic powder	1 lb	8101	Patterson Dental
Dental acrylic	Dental acrylic liquid	16 oz	8502	Patterson Dental
syringe	1 ml syringe	1		
18 G needle	18 G needle	1		
Hemostat	Halsted-Mosquito Hemostat	1	13008-12	Fine Science Tools
Rat-tooth forceps	Extra Fine Graefe Forceps	1	11153-10	Fine Science Tools
Spring scissors	Student Vannas Spring Scissors	1	91500-09	Fine Science Tools
Fine forceps	Dumont #5SF Forceps	2	11252-00	Fine Science Tools
Magnetic board	QuadHands WorkBench with movable magnetic arms	1		Amazon
Magnetic arms	From QuadHands WorkBench	2		Amazon
Alligator clips	From QuadHands WorkBench	2		Amazon
Suture	Coated Vicryl Violet Braided Suture	1	J397H	Ethicon

### Data acquisition

To record diaphragm EMG signals, the data acquisition system requires a differential amplifier, an analog-to-digital conversion interface, and a computer for the control of the recording software, data visualization, and storage of raw data. The use of a digital signal processing unit can be included in the equipment configuration for real-time pre- and postprocessing of the recorded EMG signal. The data provided here used the following settings on the differential amplifier: gain of 1K and a bandpass filter with a low cutoff of 0.1 kHz and high cutoff of 5–10 kHz. The sampling rate was set to 25 kHz. At a minimum, sampling frequency should be set for at least 2.5 times the expected maximum frequency of the signal to reduce the risk of aliasing (Nyquist, 1928; Shannon, 1949; Jerri, 1977; Merletti and Muceli, 2019). Diaphragm EMG signals from the implant are carried to a commutator to enable animal movement while preventing cables from becoming tangled (6 Channel System Commutator, Part SL6C, Protech International) through a six-pin cable (6 Channel Cable with Spring, 363-363, Protech International). A second six-pin cable (6 Channel Cable with no Spring, 363-491/6, Protech International) then carries the signal to an adapter (BNC female to double biding post; A-M Systems 726034) to convert to a BNC cable with a five-pin cable connector (A-M Systems 521500) which plugs into the amplifier. EMG recordings should be stored in its raw format to avoid data loss or corruption. Readers are referred to McManus et al. and Jonkman et al. for detailed information on terminology, setup, and data acquisition of EMG signals (McManus et al., 2021; Jonkman et al., 2024). This system can be utilized in conjunction with both head-out and whole-body plethysmography (WBP rat chamber tower and WBP tower comm lid, Data Science International) to allow simultaneous recordings of diaphragm EMG and ventilatory parameters.

## Results

### Financial and clinical outcomes of diaphragm EMG implant

The diaphragm EMG implant described here costs ∼$70 to produce and takes ∼1 h to assemble. As included in Table 1, fabricating these electrodes requires standard laboratory equipment. The EMGs can be implanted in roughly 2 h or less, and as shown in Table 3, we calculated the incidence rates of surgical and postoperative complications from *n* = 105 EMG implant surgeries and provided steps to reduce adverse events associated with each complication. In our hands, adverse complications that interfere with the ability to record signals are relatively infrequent, including a 2% risk for pneumothorax and loss of diaphragm EMG signal on one side in 5% of implanted animals.

**Table 3.** Surgical and postoperative complications and incidence rates associated with implanting chronic diaphragm EMGs and suggested steps to reduce each complication

Complication	Incidence rate	Mitigating solutions
Pneumothorax/diaphragm tearing	∼2%	- Use of minimal force to advance needle - Use of diaphragm contraction to advance needle - Ensure smooth needle/wire surface(s) - Holding base of needle to thread needle and wire through diaphragm in a fluid movement
Idiopathic loss of unilateral diaphragm EMG signal	∼5%	- Use of flux during implant fabrication - Leaving slack outside the abdominal cavity to accommodate animal growth
Infection requiring additional treatment	∼3%	- Proper sterile surgical technique - Proper handling of sterile supplies - Frequent (3 times/week) bedding/cage changes following implantation due to abdominal incision - Use of Baytril and meloxicam postsurgery
Bleeding at site of headcap insertion (<24 h after implantation)	∼50%	- Resolves spontaneously without intervention
Grooming-induced opening of abdominal incision	∼5%	- Subcuticular suture technique - Use of rodent Elizabethan collar - Incidence decreases after buprenorphine treatment
Idiopathic dehiscence of incision caudal to headcap	∼2%	- Interrupted absorbable suture caudal to implant - Surgical glue to assist in wound closure

Incidence rates are based on observations from *n* = 105 surgeries, excluding the initial *n* = 10 surgeries, which were used for surgical training and optimization and refinement of the surgical approach.

### Data processing pipeline for diaphragm EMG signals

**Figure 3.**
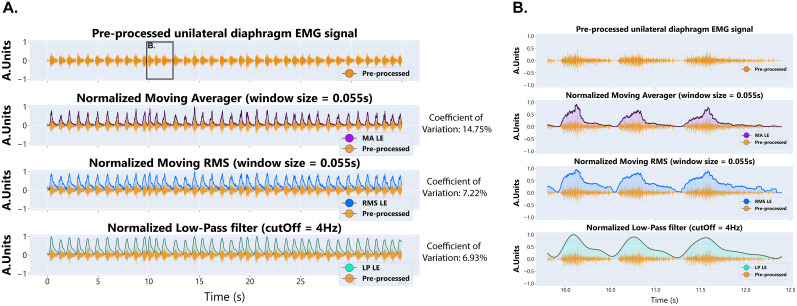
Examples of processed diaphragm EMG signals. ***A***, ***B***, A representative, preprocessed unilateral diaphragm EMG trace during eupneic breathing and examples of varied data processing approaches for the signal across a compressed (***A***) and expanded (***B***) timeline. CV was calculated from the processed data prior to normalization and is noted for each data processing approach for the compressed timeline. RMS, root mean square.

The first steps in analysis of the EMG recording are to extract the linear envelop by denoising the raw signal, rectifying, and applying a low-pass filter to the raw diaphragm EMG signal. Traditionally, this has been performed by using a moving average filter, moving root mean square (RMS), or a low-pass filter. To facilitate data analysis of diaphragm EMG signals collected with this implant, we have provided a Python code to extract the linear envelope by each of these methods (Data Repository 2 https://figshare.com/s/c04c07ecf06424e4e56a). NEO (Garcia et al., 2014) was used to load the data efficiently from CED data files. Examples of raw and processed diaphragm EMG signals using this data pipeline are shown in Figure 3. We calculated the CV of the peak EMG amplitude for each processing method and show that the CV for each method (normalized moving average, 14.75%; normalized moving RMS, 7.22%; normalized low-pass filter, 6.93%) aligns with published CV from diaphragm EMG (Mantilla et al., 2011). The instructions for key steps, a link to GitHub, and annotations of the original code used throughout the pipeline for EMG signal integration are provided in the linked repository. Additional programs may be needed for more sophisticated analyses or handling significantly long recordings. It is important to note that each of these approaches is susceptible to noise, movement artifact, and electrocardiogram (ECG) artifact given the location of the diaphragm muscle relative to the heart. Specifically, we see a more prominent ECG signal on the left hemidiaphragm EMG recording given the proximity of the electrode to the cardiac tissue. In our experience, the ECG signal is position dependent and is more prominent when animals are in a crouched/flexed or resting position as compared with locomotive and upright positions.

### Diaphragm EMG signals during ventilatory and nonventilatory behaviors

**Figure 4.**
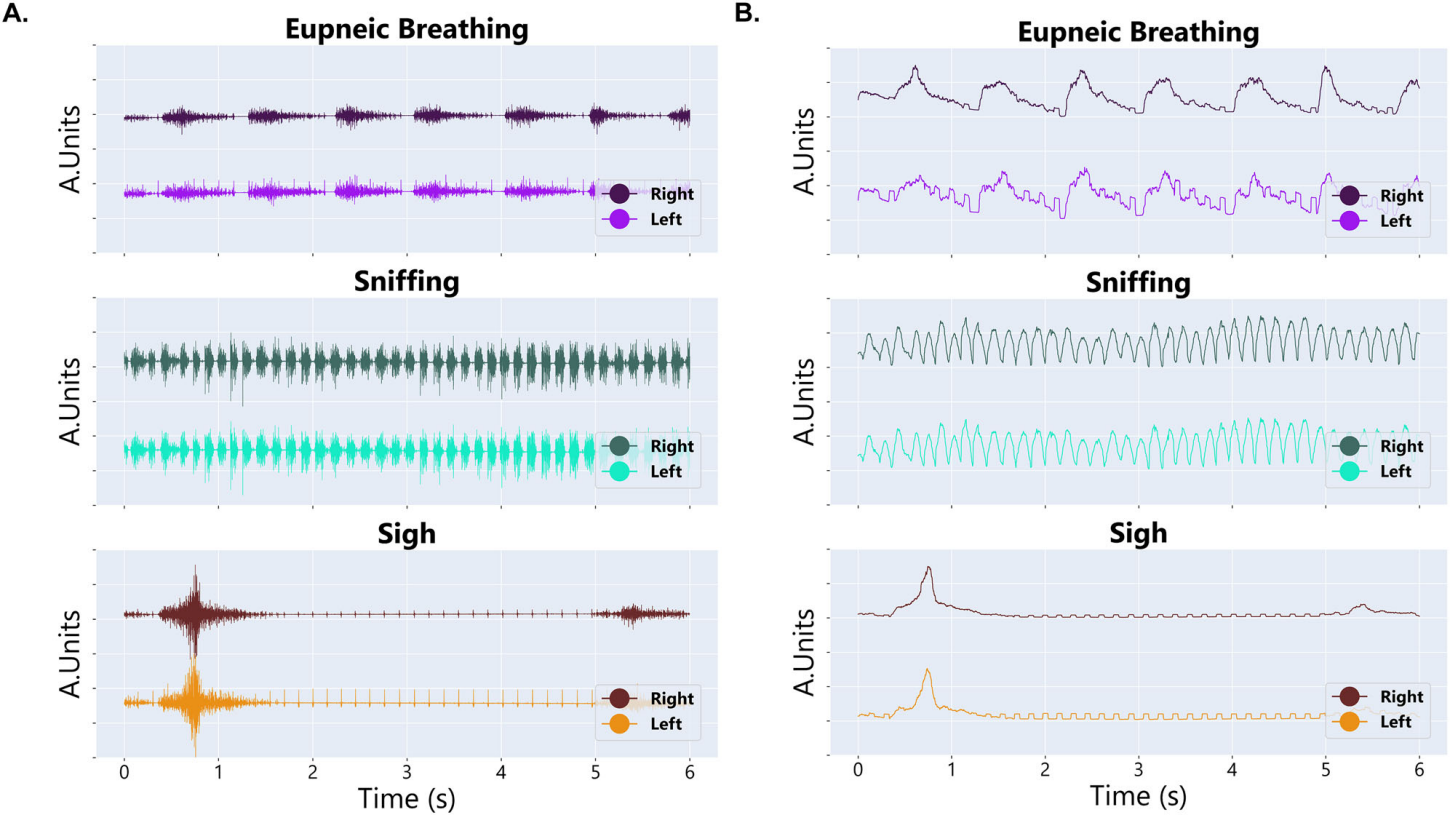
Diaphragm EMG signals across respiratory behaviors. ***A***, Representative raw bilateral diaphragm EMG traces for an awake, *ad libitum* behaving rodent across ventilatory and nonventilatory behaviors using the described implant. ***B***, Integrated bilateral diaphragm EMG signals using the provided data pipeline for an awake, *ad libitum* behaving rodent across ventilatory and nonventilatory behaviors.

**Figure 5.**
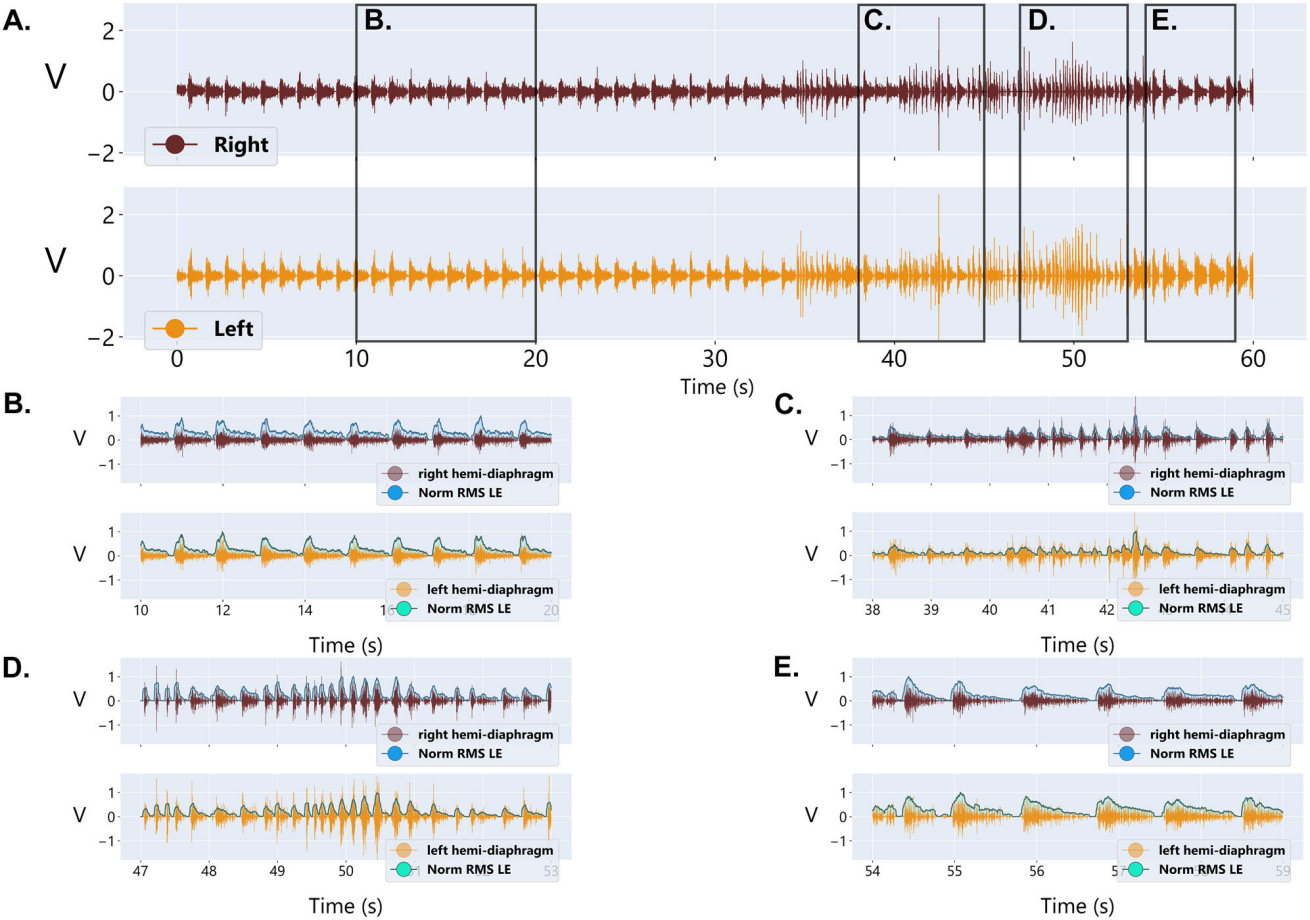
Bilateral diaphragm EMG across respiratory behaviors in an awake, *ad libitum* behaving rodent. ***A***, Representative diaphragm EMG traces from the left and right hemidiaphragms during a 1 min recording in a *ad libitum* behaving animal showing various behaviors including (***B***) quiet breathing, (***C***) breathing during locomotion, (***D***) sniffing, and (***E***) return to quiet breathing.

Using the provided data pipeline, we show representative raw and integrated EMG recordings from the left and right hemidiaphragm in awake, *ad libitum* behaving animals (Figs. 4, 5). Specifically, we provide expanded traces to show both ventilatory and nonventilatory behaviors including eupneic breathing, sniffing, and a sigh (Fig. 4). A distinct waveform is observed in the raw diaphragm EMG during each behavior. In addition, we include an example trace of diaphragm EMG activity taken from an animal while in their cage showing transitions between quiet resting breathing, breathing during locomotion, and sniffing (Fig. 5). These data show the ability of our implanted electrodes to capture both ventilatory and nonventilatory behaviors during rest and active locomotion.

### Stability of diaphragm EMG signals

**Figure 6.**
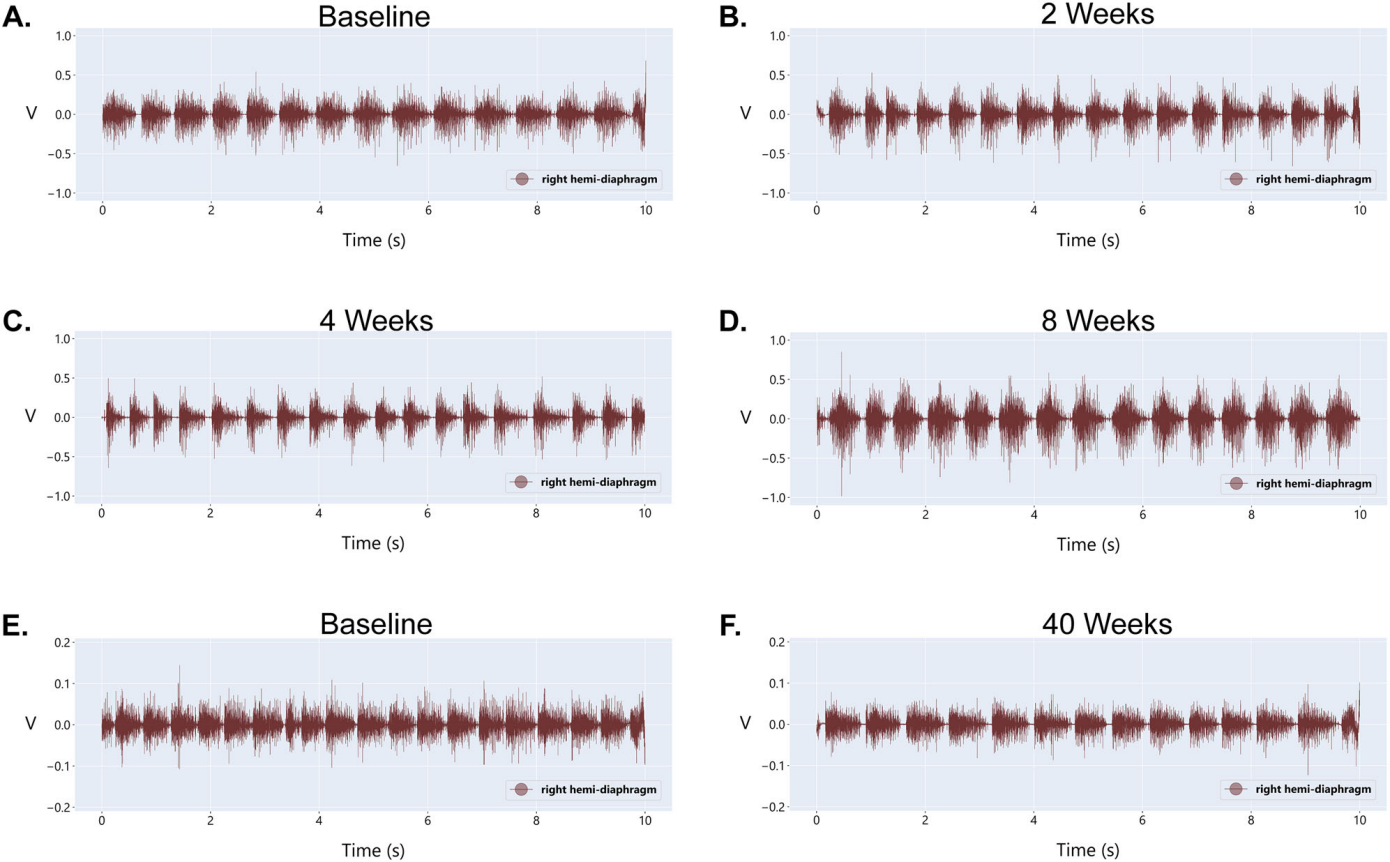
Diaphragm EMG recordings over prolonged time periods. Representative diaphragm EMG recordings from (***A***) the baseline, (***B***) 2 weeks postimplant, (***C***) 4 weeks postimplant, and (***D***) 8 weeks postimplant. Representative diaphragm EMG recordings from a subset of animals have been collected during (***E***) the baseline and (***F***) 40 weeks postimplant. Note: panels ***A–D*** were collected with an amplification of 1,000×, and panels ***E*** and ***F*** were collected with an amplification of 100×.

We routinely record diaphragm EMG signals for several months (Holmes et al., 2024), and signals have remained viable for at least 40 weeks following implantation (Fig. 6). As reported in Holmes et al. (2024), we did not detect differences in impedance after 8,weeks in male or female rats. To further examine the stability of these electrodes, we calculated the signal-to-noise ratio and did not detect differences across 8,weeks (*n* = 13; *p* = 0.4551, paired *t* test) or 40,weeks (*n* = 6; *p* = 0.4357, paired *t* test) postimplant. We also determined the CV for peak EMG amplitude during resting breathing 4–5 d after surgery and show a range of 4.5–14.4% with a mean (±SEM) of 7.04 ± 0.73% (*n* = 13). Across 8,weeks, intra-animal CV ranged from 3.1 to 7.1% with a mean of 4.803 ± 0.35% (*n* = 13). These data support the long-term stability of our electrodes to capture diaphragm EMG signals for recordings over several months.

### Simultaneous measurements of plethysmography

**Figure 7.**
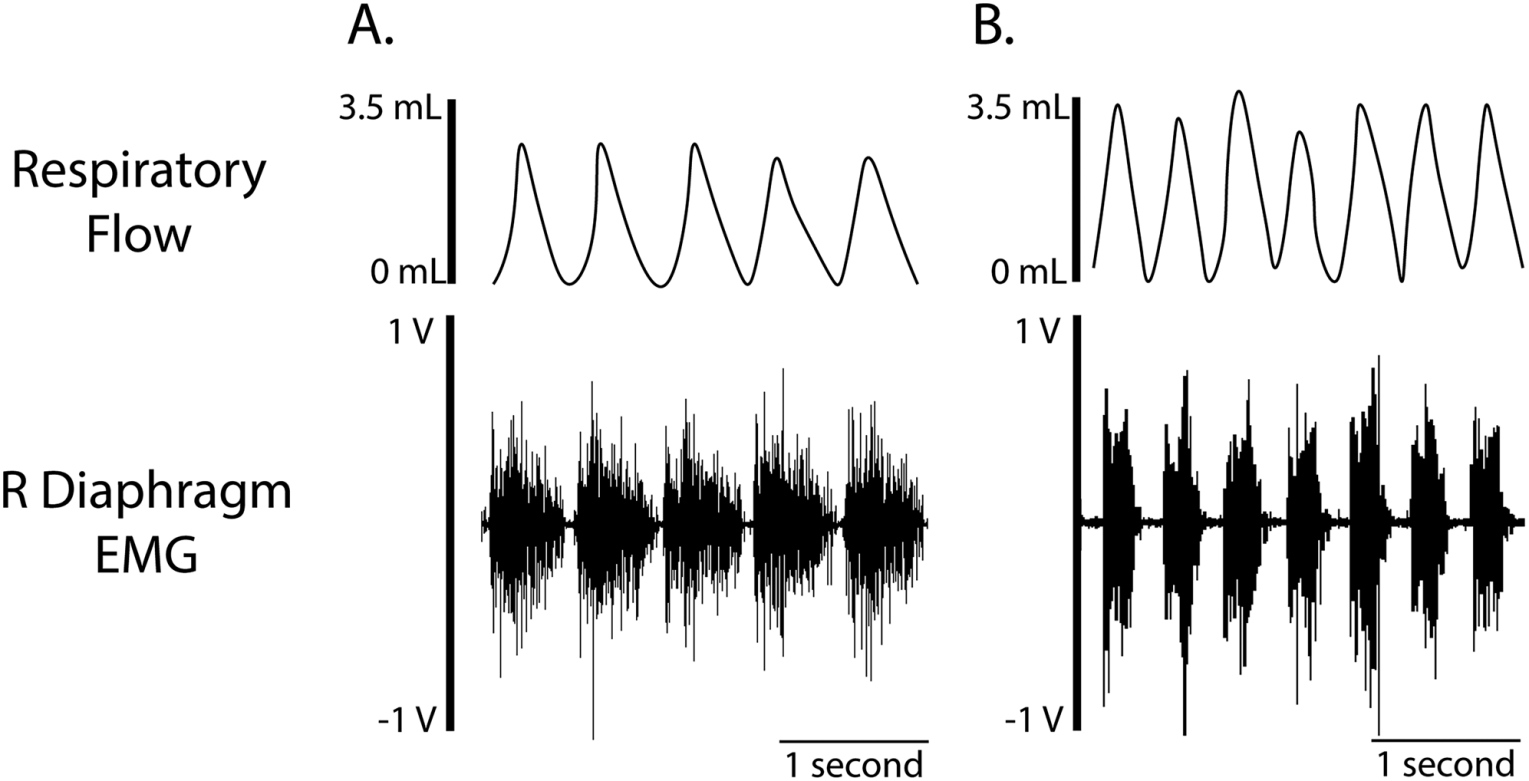
Simultaneous collection of diaphragm EMG and whole-body plethysmography. Representative respiratory flow and right diaphragm EMG traces during (***A***) normoxia and (***B***) a maximal hypoxia (10.5%) and hypercapnia (7%) challenge.

Using this approach, animals can be placed in a modified whole-body plethysmography chamber to simultaneously collect multiple respiratory variables. The modified plethysmography chambers use a commutator to facilitate animal movement and prevent cables from becoming tangled. We provide an example of simultaneous recordings of diaphragm EMG output and whole-body plethysmography during normoxia and a maximal respiratory challenge (Fig. 7). As expected, during respiratory challenge, both tidal volume and diaphragm EMG output are increased.

## Discussion

Diaphragm EMG recordings have been essential in building an understanding of respiratory neuromotor control (Sieck and Fournier, 1990; Goshgarian, 2009; Mantilla et al., 2010; Seven et al., 2014; Del Negro et al., 2018). Recording diaphragm activity in awake, *ad libitum* behaving animals is crucial to deepen our understanding of basic respiratory function and improve translation of preclinical data. This approach eliminates the confounding effects of anesthesia, allows for nonventilatory but physiologically important behaviors to be captured, facilitates longitudinal studies of diaphragm EMG signals, and enables simultaneous collection of ventilatory data via plethysmography. While telemetry-based EMG systems compatible with the diaphragm muscle are commercially available, these systems do not allow investigators to adjust recording parameters, may have sampling limitations, have limited battery life and unknown rates of success, and carry significant costs. Therefore, we provide detailed instructions on preparing and implanting affordable diaphragm EMGs for chronic recordings in awake animals. The diaphragm EMG implant detailed here costs ∼$70, takes about an hour to assemble, and uses standard laboratory equipment to produce. In addition, the procedures needed to implant these EMGs carry a minimal risk for adverse events and can be implanted in roughly 2 h or less. Although these methods were developed for the diaphragm, our group has also had success in other respiratory muscles such as the geniohyoid (Keilholz et al., 2023), external intercostals, and genioglossus (unpublished observations).

We selected stainless steel for our EMG wires due to its mechanical stability, resistance to corrosion (Bekmurzayeva et al., 2018), and previous success (Dow et al., 2006; Mantilla et al., 2011; Malone et al., 2022; Popp et al., 2023; Holmes et al., 2024; Mickle et al., 2024). Given the propensity of stainless steel electrodes for biofilm formation (Bekmurzayeva et al., 2018), monitoring the signal-to-noise ratio and impedance can be performed as an index of the tissue–electrode interface (Cogan, 2008; Gaire et al., 2018; Mierzejewski et al., 2020; O’Sullivan et al., 2024). In our hands, electrode impedance is stable across days (Malone et al., 2022) and months (Holmes et al., 2024) postimplant. Additionally, we provide new data that suggest our approach provides a high signal-to-noise ratio which does not significantly change over time. Finally, our data show that chronic diaphragm EMGs have a low CV over time. Together these measurements suggest this approach produces stable and reliable diaphragm EMG signals.

Due to the proximity of the diaphragm to the heart, ECG signals can also be present in the diaphragm EMG recordings. While ECG signals can be removed from EMG traces by a variety of methods (Taelman et al., 2007; Zhan et al., 2010; Nitzken et al., 2013; Estrada et al., 2017; Jonkman et al., 2021; Liang et al., 2023; Wang et al., 2023) or through the purchase of commercially available ECG-blankers, this removal may not be necessary given the regularity of ECG burst shape, frequency, and amplitude (Jensen et al., 2017). To date, we have had success handling ECG and other “noise” in our EMG recordings through the use of low-/high-/bandpass filters, notch filters, and comb filters. Additionally, there is substantial published work highlighting methods to mitigate the noise captured through EMG recordings (Lijun, 2005; Boyer et al., 2023; Halidou et al., 2023). The provided data processing pipeline offers a basic entry point for the transition of raw recordings to the linear envelop. While a formal discussion regarding pre- and postprocessing of data is beyond the scope of this manuscript, the reader is referred to recent literature reviews for more in-depth discussions on methodologies (Carvalho et al., 2023; Jonkman et al., 2024).

While not currently used by our group, the use of hydrogel-based adhesives may offer an advantage for implanting the electrodes by reducing tissue damage resulting from insertion of the needle (Zhou et al., 2023). Furthermore, the potential use of tissue adhesives may aid in placement of electrodes without compromising the muscle membrane and offer promise for surgical closure of tissue layers (Yuk et al., 2019). Advancements in materials science present an opportunity to optimize electrode design with improved impedance responses through the use of metal–polymer compositions and increased channel density to allow for a more in-depth study of the motor unit. For example, the Myomatrix electrode by the CAMBER group is a multichannel electrode that allows for high-density recordings of muscle activity via a flexible probe (Chung et al., 2023). Similar systems can be applied to the diaphragm muscle and enable more in-depth investigations into respiratory neuromuscular control in both healthy and pathological conditions.

Diaphragm EMG recordings from awake animals can offer important insights and informative data for understanding respiratory neuromuscular control. It should be noted that the EMG signal does not explain the mechanical aspects of contraction and only reflects the electrical events of a muscle contraction (Roberts and Gabaldón, 2008). Collectively, the data provided here shows the utility of this method to collect multiple outcome measures in each animal over long experimental timelines. This approach is particularly useful to characterize breathing deficits and potential therapeutic treatments during diseases such as spinal cord injury, Pompe disease, Duchenne muscular dystrophy, or other conditions in which breathing may be impaired. For example, we have used this method to simultaneously examine the bilateral diaphragm EMG output and ventilation during normoxia and respiratory challenge following spinal cord injury (Holmes et al., 2024). Additionally, when implanted before spinal cord injury, the implanted wires can be used to verify or validate functional deficits during and immediately after the injury. Diaphragm EMG activity can also be used to classify basal breathing from nonventilatory behaviors such as sniffing through analysis of EMG burst frequency (Moore et al., 2013; Bezdudnaya et al., 2018) or with approaches such as machine learning. Recently, Obaid and colleagues provided a model which incorporates multiple components of the diaphragm EMG burst such as respiratory cycle duration and peak amplitude to automate the identification of eupneic breathing (Khurram et al., 2024a,b). This method would be highly beneficial due to the potential to collect large volumes of data during longitudinal studies.

Common analyses applied to diaphragm EMG recordings include amplitude, time-to-peak, and onset/offset duration of the EMG signal. Amplitude assesses the magnitude of muscle activity during a contraction and can be used to determine the relative change in a recording over time. However, the ability to make comparisons across subjects or across multiple recordings is reliant on data normalization (Law et al., 2011; Mantilla et al., 2011; Jonkman et al., 2024). Several normalization methods are common, including the following: (1) to normalize diaphragm EMG amplitude relative to a sigh or “near maximal” breath (Mantilla et al., 2011; Hernandez-Torres et al., 2017; Rana et al., 2017; Seven et al., 2018), (2) to preintervention (Rana et al., 2021, 2024), or (3), in the case of spinal cord injury, to preinjury amplitude (Navarrete-Opazo et al., 2015; Rana et al., 2021; Sieck et al., 2021; Fogarty et al., 2023). These methods reduce intra-animal variability and improve the quantitative assessment of EMG signals over longitudinal studies. Time-to-peak measures the time duration from burst initiation to peak of the EMG signal and can be useful to assess neural drive (Jonkman et al., 2024). For example, calculating the RMS value of the diaphragm EMG signal 75 ms (RMS_75_) after the onset of activity can be used to estimate respiratory neural drive (Seven et al., 2014). Finally, onset/offset duration can be used to determine the duration of muscle activation during a contraction or throughout a recording period (Jonkman et al., 2024). Importantly, no clear definition exists for EMG onset/offset given that muscle activation is a multifactorial, integrated, and dynamic process. Thus, identifying the onset/offset is dependent on either automated (Roesthuis et al., 2020; Rodrigues et al., 2021) or manual (Koopman et al., 2018; Epiu et al., 2021) thresholding techniques and varies widely across labs. Examples of EMG data analysis can be found for both anesthetized (Seven et al., 2013, 2014; Gill et al., 2015; Lee and Hsu, 2017; Rana et al., 2017; Khurram et al., 2022) and awake (Bezdudnaya et al., 2018; Jimenez-Ruiz et al., 2018; Rana et al., 2021; Khurram et al., 2023) recordings.

Given the diaphragm muscle's unique characteristics, such as rhythmic contraction and the risk of pneumothorax, careful consideration of electrode materials and surgical procedures can improve the success of implanting diaphragm EMGs. The methods and resources provided here allow the scientific community to produce affordable diaphragm EMG implants that consistently produce reliable and stable signals in awake, behaving animals. The use of diaphragm EMGs during wakefulness offers an avenue for enhanced understanding of respiratory neuromuscular control and improved translation of preclinical studies.

## Synthesis

Reviewing Editor: Mark Sheffield, University of Chicago Division of the Biological Sciences Decisions are customarily a result of the Reviewing Editor and the peer reviewers coming together and discussing their recommendations until a consensus is reached. When revisions are invited, a fact-based synthesis statement explaining their decision and outlining what is needed to prepare a revision will be listed below. The following reviewer(s) agreed to reveal their identity: Obaid Khurram.

The manuscript describes a novel, low-cost implantable system for recording diaphragmatic EMG signals in awake, freely behaving rats, offering an accessible and innovative tool for respiratory neuroscience research. Both reviewers commend the methodological approach and recognize its potential value for facilitating longitudinal studies in respiratory function without the confounding effects of anesthesia.

However, the reviewers raise important concerns about the manuscript's presentation of the method's reliability, robustness, and broader applicability. Reviewer 1 emphasizes the need for quantitative evidence, such as statistical analyses, to convincingly demonstrate the stability and reliability of the recorded EMG signals over time, particularly given the manuscript's reliance on representative figures. Reviewer 2 supports the study's value but notes that it would benefit from additional methodological details, especially regarding data acquisition parameters and signal processing steps, to ensure reproducibility.

Both reviewers recommend that the authors expand the discussion of potential limitations, particularly regarding challenges such as signal artifacts and complications arising during implantation. Including more detailed comparisons to alternative methods and a broader perspective on the method's future applications would also enhance the manuscript's impact.

Overall, with revisions that address these key points-ensuring methodological rigor and clarity-this manuscript has the potential to make a valuable contribution to the field. The specific comments from both reviewers provided below should guide the authors in addressing these aspects in their revision.

Reviewer 1:

This report, "A simple, low-cost implant for reliable diaphragm EMG recordings in awake, freely behaving rats" describes methods for fabricating and implanting indwelling diaphragmatic EMG electrodes that allow recordings from awake rats. While the cost is a benefit of their approach, it is more important to disseminate reliable and rigorous approaches to the community. I do not doubt the quality of the authors' approach, but it is important that the report convincingly communicate the reliability, performance, and rigor of the approach through statistical analysis rather than the provision of example or representative figures. Additionally, the manuscript would benefit further discussion/consideration in limitations. If these weaknesses are appropriately addressed, this approach could be widely cited, adopted and impactful. Specific points are made below.

Several aspects for the quality of performance should be demonstrated in the results provided. The reader would benefit on demonstrating the reliability and robustness of their EEG recording methodology. Statistical comparisons signal quality with each example described above should be included.

• To demonstrate reliability, a comparison of cycle-to-cylce raw EEG or RMS values of individual breaths from a recording period would nice rather than individual just representative windows.

• To demonstrate accuracy, a figure where simultaneous measurements of Tidal Volume using whole-body plethysmography and EEG activity are made would be nice for the reader. This would provide an opportunity to compare variability between two different approaches and potentially, illustrate the advantage of the EEG approach when in terms of movement artifacts.

• To demonstrate short-term (hours/minute) stability and robustness of the approach, large-scale time window showing the performance of the methods when animals are actively locomoting and the transition to quiet states is advised. It is recommended comparison(s) of signals before, during and following periods of locomotion be included.

• The authors mention recording up to 40 weeks but do not provide an example. This is a major point of their approach. To demonstrate long-term (hours/minute) stability and robustness of the approach, longitudinal performance (over the course of days and/or weeks) should be demonstrated and discussed. It is recommended comparison(s) of signals over the time window be included.

• The utility of the approach should be demonstrated. For example, recordings of left-right diaphragmatic activity would be great to see for readers who may be interested in spinal cord injury.

The authors should describe limitations and/or pitfalls they are aware of with the approach and how they dealt with it.

• For example, in some traces there appears to be artifact due to the cardiac waveform. While this may not be an issue, control measurements, such artifact may complicate recordings where spontaneous diaphragmatic fasciculations may occur such as ALS, fentanyl-induced wood chest syndrome and/or seizure. The authors should describe how one may be able to minimize such artifacts or discuss such potential limitations.

• The authors mention that they observed minimal risk of pneumothorax (~2%, unpublished observations). How many animals were assessed? Similarly, can the authors describe the rates of spontaneous or accidental (due to implantation hook) diaphragmatic tearing and the occurrence of infection they observed. The results section would benefit from having a table describing common/potential complications, the rate they observed such complications and how they potentially mitigated it.

• In the discussion, consider expanding on how this method could be adapted or improved in the future, such as using different materials or refining surgical techniques.

Reviewer 2:

General Comments

This manuscript describes the development and application of an affordable, durable implant for recording diaphragm EMG signals in awake, freely behaving rats. This is an important advancement for the field, as it enables longitudinal studies of diaphragm function without the need for anesthesia, which often confounds results. The proposed system seems to be effective in capturing high-quality, reproducible diaphragm EMG data and offers potential for studies involving both ventilatory and non-ventilatory behaviors. This manuscript will make a significant contribution to the literature. However, several areas require attention to improve clarity, experimental rigor, and data interpretation.

Specific Comments

Introduction, Line 35: The diaphragm muscle is usually divided into the costal, crural, and sternal regions.

Introduction, Line 40: Not just to "sustain inspiration" as highlighted above.

Introduction, Line 46: Scholarship--at least two of these include data from awake rats.

Introduction, Line 51: Trelease, 1982 was performed in cats

Methods, Line 317: (Major) This is a Methods paper, and as such, it should stand on its own. Methodological details on data acquisition, including relevant sampling rates and analog and digital filtering should be specified.

Methods, Line 327: (Major) The manuscript references a Python-based pipeline for data extraction and processing but lacks detailed specifications of its functionalities. Providing an overview of key steps (e.g., filtering parameters, signal rectification methods) would benefit readers aiming to replicate or adapt the pipeline. Furthermore, Python struggles when dealing with large files (e.g. high sampling rate EMG).

Discussion, Line 348: There are issues with telemetry beyond the cost, such as low sampling rates. These limitations to telemetry as an alternative are arguably a far more problematic issue than cost as an alternative should be discussed.

Discussion, Line 354-358: Is sniffing really a "ventilatory" behavior? Is "eupneic" breathing defined based on the Bezdudnaya, 2018 paper criteria based on respiratory rates? Janke et al. 2022, Sunshine et al. 2021 and other modern techniques using machine learning approaches show much greater promise and should be considered.

Discussion, Line 370: The CAMBER group electrodes are multielectrode arrays that can allow single motor unit recordings. The comparison between your electrode setup and theirs without adequately describing the differences is not entirely fair.

Minor

Units should consistently have spaces before them (e.g., "5 mm" instead of "5mm").

## References

[B1] Alvarez-Argote S, Gransee HM, Mora JC, Stowe JM, Jorgenson AJ, Sieck GC, Mantilla CB (2016) The impact of midcervical contusion injury on diaphragm muscle function. J Neurotrauma 33:500–509. 10.1089/neu.2015.4054 26413840 PMC4779319

[B2] Ballók B, Schranc Á, Tóth I, Somogyi P, Tolnai J, Peták F, Fodor GH (2023) Comparison of the respiratory effects of commonly utilized general anaesthesia regimes in male Sprague-Dawley rats. Front Physiol 14:1249127. 10.3389/fphys.2023.1249127 37791348 PMC10544940

[B3] Bekmurzayeva A, Duncanson WJ, Azevedo HS, Kanayeva D (2018) Surface modification of stainless steel for biomedical applications: revisiting a century-old material. Mater Sci Eng C Mater Biol Appl 93:1073–1089. 10.1016/j.msec.2018.08.049 30274039

[B4] Bezdudnaya T, Hormigo KM, Marchenko V, Lane MA (2018) Spontaneous respiratory plasticity following unilateral high cervical spinal cord injury in behaving rats. Exp Neurol 305:56–65. 10.1016/j.expneurol.2018.03.014 29596845 PMC5955798

[B5] Boyer M, Bouyer L, Roy JS, Campeau-Lecours A (2023) Reducing noise, artifacts and interference in single-channel EMG signals: a review. Sensors 23:6. 10.3390/s23062927 36991639 PMC10059683

[B6] Carvalho CR, Fernández JM, Del-Ama AJ, Oliveira Barroso F, Moreno JC (2023) Review of electromyography onset detection methods for real-time control of robotic exoskeletons. J Neuroeng Rehabil 20:141. 10.1186/s12984-023-01268-8 37872633 PMC10594734

[B7] Chung B, et al. (2023) Myomatrix arrays for high-definition muscle recording. Elife 12:1–21. 10.7554/eLife.88551 38113081 PMC10730117

[B8] Cogan SF (2008) Neural stimulation and recording electrodes. Annu Rev Biomed Eng 10:275–309. 10.1146/annurev.bioeng.10.061807.160518 18429704

[B9] Del Negro CA, Funk GD, Feldman JL (2018) Breathing matters. Nat Rev Neurosci 19:351–367. 10.1038/s41583-018-0003-6 29740175 PMC6636643

[B10] Dow DE, Mantilla CB, Zhan WZ, Sieck GC (2006) EMG-based detection of inspiration in the rat diaphragm muscle. Conf Proc IEEE Eng Med Biol Soc 2006:1204–1207. 10.1109/iembs.2006.260688 17946030

[B11] Dow DE, Zhan WZ, Sieck GC, Mantilla CB (2009) Correlation of respiratory activity of contralateral diaphragm muscles for evaluation of recovery following hemiparesis. Annu Int Conf IEEE Eng Med Biol Soc 2009:404–407. 10.1109/iembs.2009.5334892 19965125 PMC3898802

[B12] Emmer KM, Celeste NA, Bidot WA, Perret-Gentil MI, Malbrue RA (2019) Evaluation of the sterility of Press’n seal cling film for use in rodent surgery. J Am Assoc Lab Anim Sci 58:235–239. 10.30802/aalas-jaalas-18-000096 30813984 PMC6433354

[B13] Epiu I, Gandevia SC, Boswell-Ruys CL, Basha C, Archer SNJ, Butler JE, Hudson AL (2021) Inspiratory muscle responses to sudden airway occlusion in chronic obstructive pulmonary disease. J Appl Physiol 131:36–44. 10.1152/japplphysiol.00017.2021 33955264

[B14] Estrada L, Torres A, Sarlabous L, Jané R (2017) Influence of parameter selection in fixed sample entropy of surface diaphragm electromyography for estimating respiratory activity. Entropy 19:460. 10.3390/e19090460

[B15] Fogarty MJ, Mantilla CB, Sieck GC (2018) Breathing: motor control of diaphragm muscle. Physiology 33:113–126. 10.1152/physiol.00002.2018 29412056 PMC5899234

[B16] Fogarty MJ, Zhan WZ, Simmon VF, Vanderklish PW, Sarraf ST, Sieck GC (2023) Novel regenerative drug, SPG302 promotes functional recovery of diaphragm muscle activity after cervical spinal cord injury. J Physiol 601:2513–2532. 10.1113/jp284004 36815402 PMC10404468

[B17] Fuller DD, Rana S, Smuder AJ, Dale EA (2022) The phrenic neuromuscular system. Handb Clin Neurol 188:393–408. 10.1016/b978-0-323-91534-2.00012-6 35965035 PMC11135908

[B18] Gaire J, Lee HC, Hilborn N, Ward R, Regan M, Otto KJ (2018) The role of inflammation on the functionality of intracortical microelectrodes. J Neural Eng 15:066027. 10.1088/1741-2552/aae4b6 30260321

[B19] Garcia S, et al. (2014) Neo: an object model for handling electrophysiology data in multiple formats. Front Neuroinform 8:10. 10.3389/fninf.2014.00010 24600386 PMC3930095

[B20] Gill LC, Mantilla CB, Sieck GC (2015) Impact of unilateral denervation on transdiaphragmatic pressure. Respir Physiol Neurobiol 210:14–21. 10.1016/j.resp.2015.01.013 25641347 PMC4449269

[B21] Goshgarian HG (2009) The crossed phrenic phenomenon and recovery of function following spinal cord injury. Respir Physiol Neurobiol 169:85–93. 10.1016/j.resp.2009.06.005 19539790 PMC2783917

[B22] Halidou A, Mohamadou Y, Ari AAA, Zacko EJG (2023) Review of wavelet denoising algorithms. Multimed Tools Appl 82:41539–41569. 10.1007/s11042-023-15127-0

[B23] Hernandez-Torres V, Gransee HM, Mantilla CB, Wang Y, Zhan WZ, Sieck GC (2017) BDNF effects on functional recovery across motor behaviors after cervical spinal cord injury. J Neurophysiol 117:537–544. 10.1152/jn.00654.2016 27832605 PMC5288474

[B24] Holmes TC, Popp NM, Hintz CF, Dobrzycki I, Schmitz CJ, Schwichtenberg KA, Gonzalez-Rothi EJ, Sundberg CW, Streeter KA (2024) Sex differences in spontaneous respiratory recovery following chronic C2 hemisection. J Appl Physiol 137:166–180. 10.1152/japplphysiol.00040.2024 38867665 PMC11381122

[B25] Hunter JD, Milsom WK (1998) Cortical activation states in sleep and anesthesia. I: cardio-respiratory effects. Respir Physiol 112:71–81. 10.1016/s0034-5687(98)00018-8 9696284

[B26] Jensen VN, Romer SH, Turner SM, Crone SA (2017) Repeated measurement of respiratory muscle activity and ventilation in mouse models of neuromuscular disease. J Vis Exp 122:1–9. 10.3791/55599 28448001 PMC5565023

[B27] Jerri AJ (1977) The Shannon sampling theorem—its various extensions and applications: a tutorial review. Proc IEEE 65:1565–1596. 10.1109/PROC.1977.10771

[B28] Jimenez-Ruiz F, Khurram OU, Zhan WZ, Gransee HM, Sieck GC, Mantilla CB (2018) Diaphragm muscle activity across respiratory motor behaviors in awake and lightly anesthetized rats. J Appl Physiol 124:915–922. 10.1152/japplphysiol.01004.2017 29357493 PMC5972468

[B29] Jonkman AH, et al. (2024) Analysis and applications of respiratory surface EMG: report of a round table meeting. Crit Care 28:2. 10.1186/s13054-023-04779-x 38166968 PMC10759550

[B30] Jonkman AH, Juffermans R, Doorduin J, Heunks LMA, Harlaar J (2021) Estimated ECG subtraction method for removing ECG artifacts in esophageal recordings of diaphragm EMG. Biomed Signal Process Control 69:102861. 10.1016/j.bspc.2021.102861

[B31] Keilholz A, et al. (2023) Tongue exercise-induced functional and structural upper airway plasticity in a rodent model of hypoglossal (XII) motor neuron loss. Physiology 38:5729638. 10.1152/physiol.2023.38.S1.5729638

[B33] Khurram OU, Gransee HM, Sieck GC, Mantilla CB (2022) Automated evaluation of respiratory signals to provide insight into respiratory drive. Respir Physiol Neurobiol 300:103872. 10.1016/j.resp.2022.103872 35218924 PMC9157394

[B34] Khurram OU, Mantilla CB, Sieck GC (2023) Neuromotor control of spontaneous quiet breathing in awake rats evaluated by assessments of diaphragm EMG stationarity. J Neurophysiol 130:1344–1357. 10.1152/jn.00267.2023 37877195 PMC11918285

[B32] Khurram OU, Fogarty MJ, Zhan W-Z, Mantilla CB, Sieck GC (2024a) Assessing functional recovery of eupneic diaphragm activity following unilateral cervical spinal cord injury in rats. J Vis Exp 208:e66828. 10.3791/66828 38949318 PMC11792163

[B35] Khurram OU, Mantilla CB, Sieck GC (2024b) Identification of eupneic breathing using machine learning. J Neurophysiol 132:678–684. 10.1152/jn.00230.2024 39052237 PMC11427058

[B36] Kocjan J, Adamek M, Gzik-Zroska B, Czyżewski D, Rydel M (2017) Network of breathing. Multifunctional role of the diaphragm: a review. Adv Respir Med 85:224–232. 10.5603/arm.2017.0037 28871591

[B37] Koopman AA, Blokpoel RGT, van Eykern LA, de Jongh FHC, Burgerhof JGM, Kneyber MCJ (2018) Transcutaneous electromyographic respiratory muscle recordings to quantify patient-ventilator interaction in mechanically ventilated children. Ann Intensive Care 8:12. 10.1186/s13613-018-0359-9 29362986 PMC5780334

[B38] Law LF, Krishnan C, Avin K (2011) Modeling nonlinear errors in surface electromyography due to baseline noise: a new methodology. J Biomech 44:202–205. 10.1016/j.jbiomech.2010.09.008 20869716 PMC3003745

[B39] Lee KZ, Hsu SH (2017) Compensatory function of the diaphragm after high cervical hemisection in the rat. J Neurotrauma 34:2634–2644. 10.1089/neu.2016.4943 28447895

[B40] Liang M, Li J, Li B, Xu Y, Mo H (2023) A modified method for attenuating ECG interference in recordings of diaphragm EMG. 2023 16th International Congress on Image and Signal Processing, BioMedical Engineering and Informatics (CISP-BMEI), 1–5.

[B41] Lijun X (2005) Cancellation of harmonic interference by baseline shifting of wavelet packet decomposition coefficients. IEEE Trans Signal Process 53:222–230. 10.1109/TSP.2004.838954

[B42] Malone IG, Kelly MN, Nosacka RL, Nash MA, Yue S, Xue W, Otto KJ, Dale EA (2022) Closed-loop, cervical, epidural stimulation elicits respiratory neuroplasticity after spinal cord injury in freely behaving rats. eNeuro 9:ENEURO.0426-21.2021. 10.1523/eneuro.0426-21.2021 35058311 PMC8856702

[B43] Mantilla CB, Greising SM, Zhan WZ, Seven YB, Sieck GC (2013) Prolonged C2 spinal hemisection-induced inactivity reduces diaphragm muscle specific force with modest, selective atrophy of type IIx and/or IIb fibers. J Appl Physiol 114:380–386. 10.1152/japplphysiol.01122.2012 23195635 PMC3568873

[B44] Mantilla CB, Seven YB, Hurtado-Palomino JN, Zhan WZ, Sieck GC (2011) Chronic assessment of diaphragm muscle EMG activity across motor behaviors. Respir Physiol Neurobiol 177:176–182. 10.1016/j.resp.2011.03.011 21414423 PMC3103648

[B45] Mantilla CB, Seven YB, Zhan WZ, Sieck GC (2010) Diaphragm motor unit recruitment in rats. Respir Physiol Neurobiol 173:101–106. 10.1016/j.resp.2010.07.001 20620243 PMC2919593

[B46] McManus L, et al. (2021) Consensus for experimental design in electromyography (CEDE) project: terminology matrix. J Electromyogr Kinesiol 59:102565. 10.1016/j.jelekin.2021.102565 34102383

[B47] Merletti R, Muceli S (2019) Tutorial. Surface EMG detection in space and time: best practices. J Electromyogr Kinesiol 49:102363. 10.1016/j.jelekin.2019.102363 31665683

[B48] Merrell AJ, Kardon G (2013) Development of the diaphragm – a skeletal muscle essential for mammalian respiration. FEBS J 280:4026–4035. 10.1111/febs.12274 23586979 PMC3879042

[B49] Mickle AR, Peñaloza-Aponte JD, Coffey R, Hall NA, Baekey D, Dale EA (2024) Closed-loop cervical epidural stimulation partially restores ipsilesional diaphragm EMG after acute C(2) hemisection. Respir Physiol Neurobiol 320:104182. 10.1016/j.resp.2023.104182 37923238 PMC11135909

[B50] Mierzejewski M, Steins H, Kshirsagar P, Jones PD (2020) The noise and impedance of microelectrodes. J Neural Eng 17:052001. 10.1088/1741-2552/abb3b4 33055360

[B51] Mills GH (2018) Respiratory complications of anaesthesia. Anaesthesia 73:25–33. 10.1111/anae.14137 29313906

[B52] Moore JD, Deschênes M, Furuta T, Huber D, Smear MC, Demers M, Kleinfeld D (2013) Hierarchy of orofacial rhythms revealed through whisking and breathing. Nature 497:205–210. 10.1038/nature12076 23624373 PMC4159559

[B53] Nason LK, Walker CM, McNeeley MF, Burivong W, Fligner CL, Godwin JD (2012) Imaging of the diaphragm: anatomy and function. Radiographics 32:E51–E70. 10.1148/rg.322115127 22411950

[B54] Navarrete-Opazo A, Mitchell GS (2014) Recruitment and plasticity in diaphragm, intercostal, and abdominal muscles in unanesthetized rats. J Appl Physiol 117:180–188. 10.1152/japplphysiol.00130.2014 24833779 PMC4101609

[B55] Navarrete-Opazo A, Vinit S, Dougherty BJ, Mitchell GS (2015) Daily acute intermittent hypoxia elicits functional recovery of diaphragm and inspiratory intercostal muscle activity after acute cervical spinal injury. Exp Neurol 266:1–10. 10.1016/j.expneurol.2015.02.007 25687551 PMC4716671

[B56] Nitzken M, Bajaj N, Aslan S, Gimel'farb G, El-Baz A, Ovechkin A (2013) Local wavelet-based filtering of electromyographic signals to eliminate the electrocardiographic-induced artifacts in patients with spinal cord injury. J Biomed Sci Eng 6:1–13. 10.4236/jbise.2013.67A2001 24307920 PMC3845519

[B57] Nyquist H (1928) Certain topics in telegraph transmission theory. Trans Am Inst Electr Eng 47:617–644. 10.1109/T-AIEE.1928.5055024

[B58] O’Sullivan KP, Philip BJ, Baker JL, Rolston JD, Orazem ME, Otto KJ, Butson CR (2024) In vivo and in vitro electrochemical impedance spectroscopy of acute and chronic intracranial electrodes. Data 9:78. 10.3390/data9060078 39916729 PMC11801193

[B59] Pickering M, Jones JF (2002) The diaphragm: two physiological muscles in one. J Anat 201:305–312. 10.1046/j.1469-7580.2002.00095.x 12430954 PMC1570921

[B60] Popp NM, Holmes TC, Streeter KA (2023) Diaphragm stimulation elicits phrenic afferent-induced neuromuscular plasticity. Respir Physiol Neurobiol 310:104014. 10.1016/j.resp.2023.104014 36642318 PMC9945879

[B61] Rana S, Sieck GC, Mantilla CB (2017) Diaphragm electromyographic activity following unilateral midcervical contusion injury in rats. J Neurophysiol 117:545–555. 10.1152/jn.00727.2016 27832610 PMC5288488

[B62] Rana S, Sunshine MD, Greer JJ, Fuller DD (2021) Ampakines stimulate diaphragm activity after spinal cord injury. J Neurotrauma 38:3467–3482. 10.1089/neu.2021.0301 34806433 PMC8713281

[B63] Rana S, Thakre PP, Fuller DD (2024) Ampakines increase diaphragm activation following mid-cervical contusion injury in rats. Exp Neurol 376:114769. 10.1016/j.expneurol.2024.114769 38582278 PMC12132906

[B64] Rana S, Zhan WZ, Mantilla CB, Sieck GC (2020) Disproportionate loss of excitatory inputs to smaller phrenic motor neurons following cervical spinal hemisection. J Physiol 598:4693–4711. 10.1113/jp280130 32735344 PMC7869015

[B65] Ricoy J, Rodríguez-Núñez N, Álvarez-Dobaño JM, Toubes ME, Riveiro V, Valdés L (2019) Diaphragmatic dysfunction. Pulmonology 25:223–235. 10.1016/j.pulmoe.2018.10.008 30509855

[B66] Roberts TJ, Gabaldón AM (2008) Interpreting muscle function from EMG: lessons learned from direct measurements of muscle force. Integr Comp Biol 48:312–320. 10.1093/icb/icn056 21669793 PMC4817590

[B67] Rodrigues A, Janssens L, Langer D, Matsumura U, Rozenberg D, Brochard L, Reid WD (2021) Semi-automated detection of the timing of respiratory muscle activity: validation and first application. Front Physiol 12:794598. 10.3389/fphys.2021.794598 35046839 PMC8762204

[B68] Roesthuis LH, van der Hoeven JG, van Hees HWH, Schellekens WM, Doorduin J, Heunks LMA (2020) Recruitment pattern of the diaphragm and extradiaphragmatic inspiratory muscles in response to different levels of pressure support. Ann Intensive Care 10:67. 10.1186/s13613-020-00684-6 32472272 PMC7256918

[B69] Seven YB, Mantilla CB, Sieck GC (2014) Recruitment of rat diaphragm motor units across motor behaviors with different levels of diaphragm activation. J Appl Physiol 117:1308–1316. 10.1152/japplphysiol.01395.2013 25257864 PMC4254843

[B70] Seven YB, Mantilla CB, Zhan WZ, Sieck GC (2013) Non-stationarity and power spectral shifts in EMG activity reflect motor unit recruitment in rat diaphragm muscle. Respir Physiol Neurobiol 185:400–409. 10.1016/j.resp.2012.08.020 22986086 PMC3529998

[B71] Seven YB, Nichols NL, Kelly MN, Hobson OR, Satriotomo I, Mitchell GS (2018) Compensatory plasticity in diaphragm and intercostal muscle utilization in a rat model of ALS. Exp Neurol 299:148–156. 10.1016/j.expneurol.2017.10.015 29056361 PMC5951687

[B72] Shannon CE (1949) Communication in the presence of noise. Proc IRE 37:10–21. 10.1109/JRPROC.1949.232969

[B73] Sieck GC, Fournier M (1989) Diaphragm motor unit recruitment during ventilatory and nonventilatory behaviors. J Appl Physiol 66:2539–2545. 10.1152/jappl.1989.66.6.2539 2745316

[B74] Sieck GC, Fournier M (1990) Changes in diaphragm motor unit EMG during fatigue. J Appl Physiol 68:1917–1926. 10.1152/jappl.1990.68.5.1917 2163376

[B75] Sieck GC, Gransee HM, Zhan WZ, Mantilla CB (2021) Acute intrathecal BDNF enhances functional recovery after cervical spinal cord injury in rats. J Neurophysiol 125:2158–2165. 10.1152/jn.00146.2021 33949892 PMC8285661

[B76] Taelman J, Van Huffel S, Spaepen A (2007) Wavelet-independent component analysis to remove electrocardiography contamination in surface electromyography. Annu Int Conf IEEE Eng Med Biol Soc 2007:682–685. 10.1109/iembs.2007.4352382 18002048

[B77] Wang KC, Liu KC, Peng SY, Tsao Y (2023) ECG artifact removal from single-channel surface EMG using fully convolutional networks. ICASSP 2023 - 2023 IEEE International Conference on Acoustics, Speech and Signal Processing (ICASSP).

[B78] Yuk H, Varela CE, Nabzdyk CS, Mao X, Padera RF, Roche ET, Zhao X (2019) Dry double-sided tape for adhesion of wet tissues and devices. Nature 575:169–174. 10.1038/s41586-019-1710-5 31666696

[B79] Zhan C, Yeung LF, Yang Z (2010) A wavelet-based adaptive filter for removing ECG interference in EMGdi signals. J Electromyogr Kinesiol 20:542–549. 10.1016/j.jelekin.2009.07.007 19692270

[B80] Zhou T, et al. (2023) 3D printable high-performance conducting polymer hydrogel for all-hydrogel bioelectronic interfaces. Nat Mater 22:895–902. 10.1038/s41563-023-01569-2 37322141

